# Replication of FTO Gene associated with lean mass in a Meta-Analysis of Genome-Wide Association Studies

**DOI:** 10.1038/s41598-020-61406-3

**Published:** 2020-03-19

**Authors:** Shu Ran, Zi-Xuan Jiang, Xiao He, Yu Liu, Yu-Xue Zhang, Lei Zhang, Yu-Fang Pei, Meng Zhang, Rong Hai, Gui-Shan Gu, Bao-Lin Liu, Qing Tian, Yong-Hong Zhang, Jing-Yu Wang, Hong-Wen Deng

**Affiliations:** 1 School of Medical Instruments and Food Engineering, University of Shanghai for Science and Technology, Shanghai, P.R. China; 2 Center for Genetic Epidemiology and Genomics, School of Public Health, Soochow University, Jiangsu, P.R. China; 3 Jiangsu Key Laboratory of Preventive and Translational Medicine for Geriatric Diseases, Soochow University, Jiangsu, P.R. China; 4 Department of Epidemiology and Statistics, School of Public Health, Soochow University, Jiangsu, P.R. China; 5 Beijing Gene Tangram Technology Development CO., Ltd., Beijing, P.R. China; 6 Inner Mongolia Autonomous Region Center of Health Management Service, Hohhot, P.R. China; 7 Ji Lin University, First Hospital, Changchun, P.R. China; 8 Department of Biostatistics and Data Science, Tulane University, New Orleans, Louisiana USA

**Keywords:** Computational biology and bioinformatics, Computational biology and bioinformatics, Genetics, Genetics

## Abstract

Sarcopenia is characterized by low skeletal muscle, a complex trait with high heritability. With the dramatically increasing prevalence of obesity, obesity and sarcopenia occur simultaneously, a condition known as sarcopenic obesity. Fat mass and obesity-associated (FTO) gene is a candidate gene of obesity. To identify associations between lean mass and FTO gene, we performed a genome-wide association study (GWAS) of lean mass index (LMI) in 2207 unrelated Caucasian subjects and replicated major findings in two replication samples including 6,004 unrelated Caucasian and 38,292 unrelated Caucasian. We found 29 single nucleotide polymorphisms (SNPs) in FTO significantly associated with sarcopenia (combined *p*-values ranging from 5.92 × 10^−12^ to 1.69 × 10^−9^). Potential biological functions of SNPs were analyzed by HaploReg v4.1, RegulomeDB, GTEx, IMPC and STRING. Our results provide suggestive evidence that FTO gene is associated with lean mass.

## Introduction

Sarcopenia is a complex disease described as the age-associated loss of skeletal muscle mass, strength and function impairment^1,2^. The low skeletal muscle mass will lead to many public health problems such as sarcopenia, osteoporosis and increased mortality^3,4^, especially in the elderly. Skeletal muscle is heritable with heritability estimates of 30–85% for muscle strength and 45–90% for muscle mass^5^. Although there are many genetic researches have shown some SNPs and copy number variants (CNVs) associated with lean mass^6–14^, the majority of specific genes underlying the variations in low lean body mass (LBM) are still unknown. And sarcopenia can be predicted by LMI^15^.

FTO gene is proved the association with fat mass, which contributes to human obesity^16–21^. According to many vivo studies using FTO overexpression or knockout mouse models, FTO gene can cause abnormal adipose tissues and body mass, implying a pivotal role of FTO in adipogenesis and energy homeostasis^22–25^. But the exact biological functions of this gene are unknown yet. In recent researches, FTO gene is proved the association with lean mass^22,23,26–31^. Zillikens *et al*. reported a series of SNPs of FTO associated with LBM and appendicular lean mass (ALM)^30^. In our study, we performed a GWAS to identify the associations between FTO and LMI in 2,207 unrelated Caucasians (516 men and 1,691 women). Then we replicated our findings in two replication samples, including 6,004 unrelated Caucasians and 38,292 unrelated Caucasians subjects^30^.

## Methods

### Ethic statement

This study was approved by institutional review boards of Creighton University and the University of Missouri-Kansas City. Before entering the study, all subjects provide written informed consent documents. The methods carried out in accordance with the approved study protocol.

### Discovery sample

The discovery sample consisted of 2,207 unrelated Caucasian subjects of European ancestry that were recruited in Midwestern U.S. (Kansas City, Missouri and Omaha, Nebraska). All discovery subjects completed a structured questionnaire covering lifestyle, diet, family information, medical history, etc. The inclusion and exclusion criteria for cases were described in our previous publication^32^.

### Replication sample

There were two replication samples which were performed association studies with other anthropometric phenotypes.

Replication sample 1 contains 6,004 unrelated Caucasian of European ancestry from Framingham heart study (FHS) which is a longitudinal and prospective cohort comprising >16,000 pedigree participants spanning three generations of European ancestry. Details about the FHS have reported previously^33^.

Replication sample 2 contains 38,292 unrelated Caucasian of European ancestry from 20 cohorts^30^. The details and GWAS results are from the genetic factors for osteoporosis (GEFOS) (http://www.gefos.org).

### Phenotyping

In present study, LBM and fat body mass (FBM) were measured using a dual-energy X-ray absorptiometry (DXA) scanner Hologic QDR 4500W machine (Hologic Inc., Bedford, MA, USA) that was calibrated daily. Height was obtained by using a calibrated stadiometer and weight was measured in light indoor clothing by a calibrated balance beam scale. LMI was calculated as the ratio of the sum of lean soft tissue (nonfat, non-bone) mass in whole body to square of height^34^.

### Genotyping and quality control

Genomic DNA was extracted from peripheral blood leukocytes using Puregene DNA Isolation Kit (Gentra systems, Minneapolis, MN, USA). For discovery sample, SNP genotyping with Affymetrix Genome-Wide Human SNP Array 6.0 was performed using the standard protocol recommended by the manufacturer. Fluorescence intensities were quantified using an Affymetrix array scanner 30007G. Data management and analyses were conducted using the Genotyping Command Console Software. We conducted strict quality control (QC) procedure. All subjects (n = 2,283) had a minimum call rate 95% and the final mean call rate reached a high level of 98.93%. We discarded SNPs that deviated from Hardy-Weinberg equilibrium (*p* < 0.01) and those containing a minor allele frequency (MAF) less than 0.01. Then we found 21,247 SNPs allele frequencies deviated from Hardy-Weinberg equilibrium, and additional 141,666 SNPs had MAF < 0.01. After QC, 746,709 SNPs remained in the discovery sample.

For replication sample 1, SNP genotyped using approximately 550,000 SNPs (Affymetrix 500 K mapping array plus Affymetrix 50 K supplemental array). For details of the genotyping method, please refer to FHS SHARe at NCBI dbGaP website (http://www.ncbi.nlm.nih.gov/projects/gap/cgi-bin/study.cgi?study_id=phs000007.v3.p2).

### Genotype imputation

Genotype imputation was applied to both the discovery and replication samples, with the 1000 Genomes projects sequence variants as reference panel (as of August 2010). Reference sample included 283 individuals of European ancestry.

The details of genotype imputation process had been described earlier^35^. Briefly, strand orientations between reference panel and test sample were checked before imputation, and inconsistencies were resolved by changing the test sample to reverse strand or removing the SNP from the test sample. Imputation was performed with MINIMAC^36^. Quality control was applied to impute SNPs with the following criteria: imputation r^2^ > 0.5 and MAF > 0.01. SNPs failing the QC criteria were excluded from subsequent association analyses.

### Statistical analyses

#### GWAS analysis

In discovery sample, we used the first five principal components, gender, age, age^2^ and FBM as covariates to screen for significance with the step-wise linear regression model implemented in R function stepAIC. Raw LMI values of discovery sample were adjusted by significant covariates (age, gender and FBM), and the residuals were normalized by inverse quantiles of standard normal distribution. MACH2QTL was used to perform genetic association analyses between SNPs and normalized residuals of LMI with an additive mode of inheritance.

#### Meta-analysis

Meta-analyses were performed by METAL software (https://genome.sph.umich.edu/wiki/METAL_Documentation) using the weighted fixed -effects model, which takes into account effect size and their standard errors.

The linkage disequilibrium (LD) patterns of the interested SNPs were analyzed and plotted using the Haploview program^37^ (http://www.broad.tamit.edu/mpg/haploview/).

### Functional annotation

We used HaploReg v4.1 (https://pubs.broadinstitute.org/mammals/haploreg/haploreg.php) to search for significant SNPs with functional annotations and the RegulomeDB^38^ (http://www.regulomedb.org/) program to rank potential functional roles.

To investigate the association between the identified SNP polymorphisms and the nearby gene expressions, we performed cis-eQTL analysis. We used the GTEx (https://gtexportal.org) project dataset for analysis^39^. The GTEx project was designed to establishing a sample and data resource to enable studies of the relationship among genetic variation, gene expression, and other molecular phenotypes in multiple human tissues.

We annotated gene by constructing gene interaction networks with STRING v.10 online platform (https://string-db.org/). STRING uses information based on gene co-expression, text-mining and others, to construct gene interactive networks.

## Results

**Figure 1:**
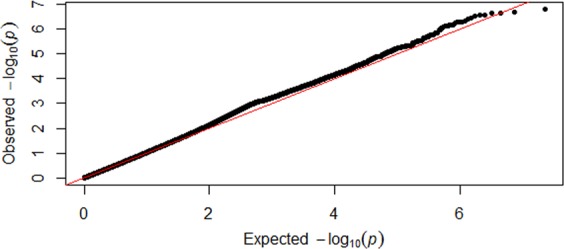
QQ plot. Logarithmic quantile–quantile (QQ) plot of individual SNP-based association for fat-adjusted LMI in the discovery sample.

**Figure 2:**
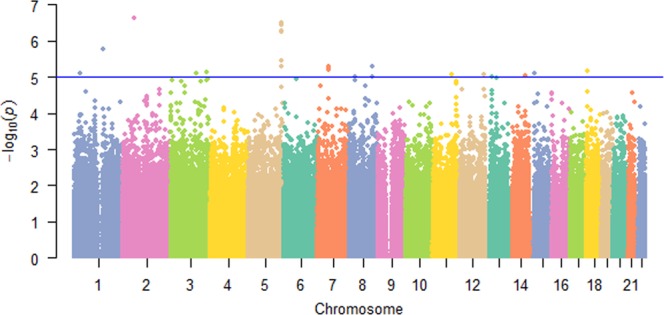
Manhattan plot of discovery GWAS samples.

Table 1 is the basic characteristics of the subjects used in discovery sample and replication sample 1. The basic characteristics of replication sample 2 are summarized in the previous research^30^. Genomic control inflation factor of discovery sample is 0.976. In order to avoid potential population stratification, we used the inflation factor to adjust individual *p*-values. Figure 1 shows the logarithmic quantile–quantile (QQ) plot of SNP-based association results. After adjustment by the genomic control approach there is no evidence of population stratification is observed. Figure 2 is Manhattan plot of the discovery sample.

**Table 1:** Basic characters of study subjects.

	Discovery sample		Replication sample 1^a^	
	Male	Female	Male	Female
Number	516	1,691	2,525	3,479
Age	51.2 (16.1)	51.7 (12.9)	54.0 (13.1)	55.9 (13.7)
Height (cm)	175.9 (7.3)	163.3 (6.3)	176.0 (7.1)	162.0 (6.8)
Weight (kg)	86.8 (16.3)	71.4 (16.0)	84.4 (13.3)	68.0 (13.8)
FBM (kg)	20.6 (9.1)	25.3 (10.8)	24.9 (9.0)	27.8 (10.5)
LBM (kg)	66.3 (9.5)	46.8 (7.0)	57.3 (7.1)	38.3 (5.2)
LMI (g/cm^2^)	2.2 (1.0)	1.8 (1.3)	1.8 (0.2)	1.5 (0.2)

Note: The numbers within parentheses are standard deviation (SD).

^a^The replication sample 1 includes 6004 unrelated Caucasian from FHS.

**Figure 3:**
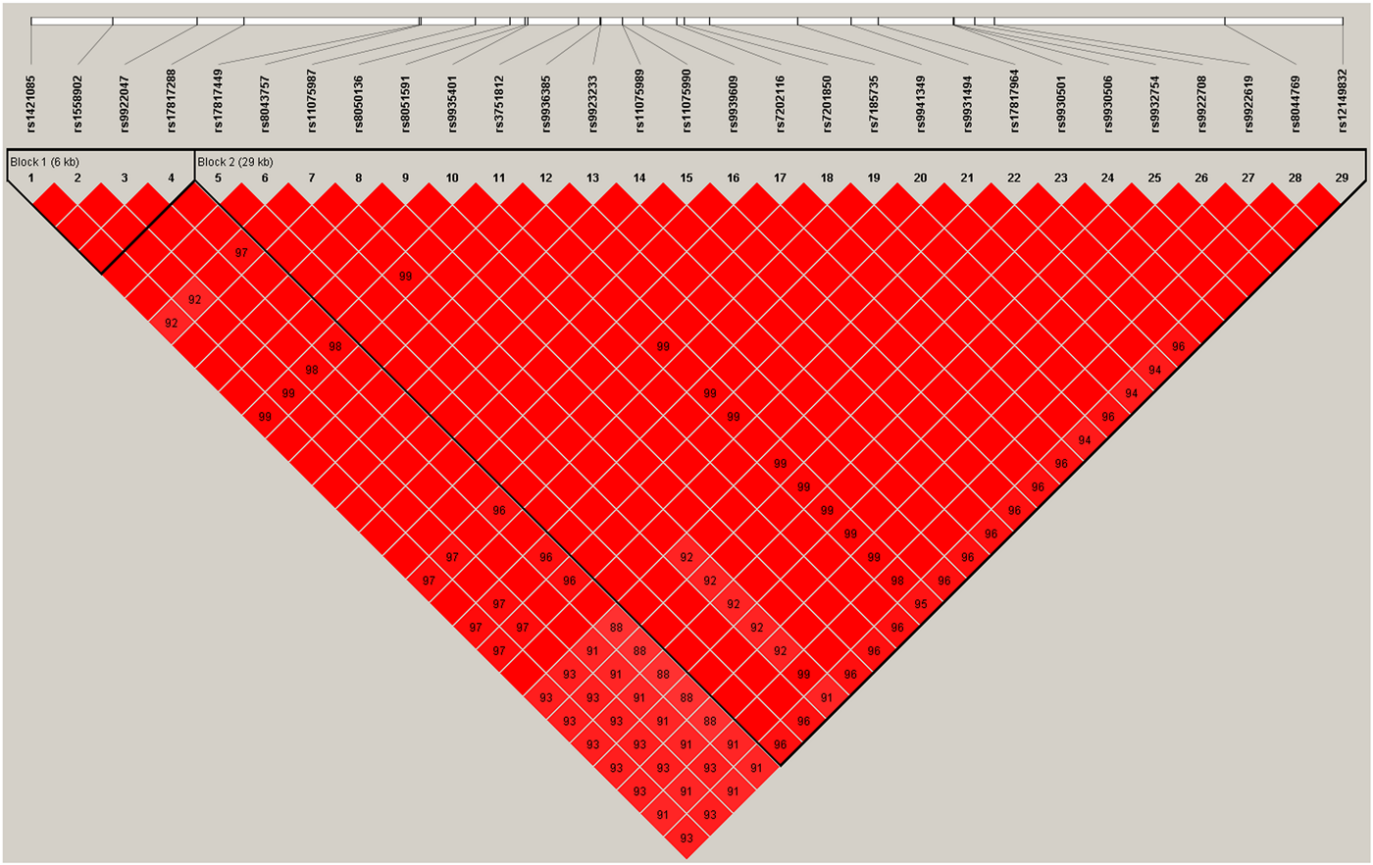
LD plot. Association signals of the 29 significant SNPs of the FTO gene. The Haploview block map for the 29 SNPs, showing pairwise LD in r^2^, was constructed for Caucasian (CEU) using the 1000 Genomes Project.

**Figure 4:**
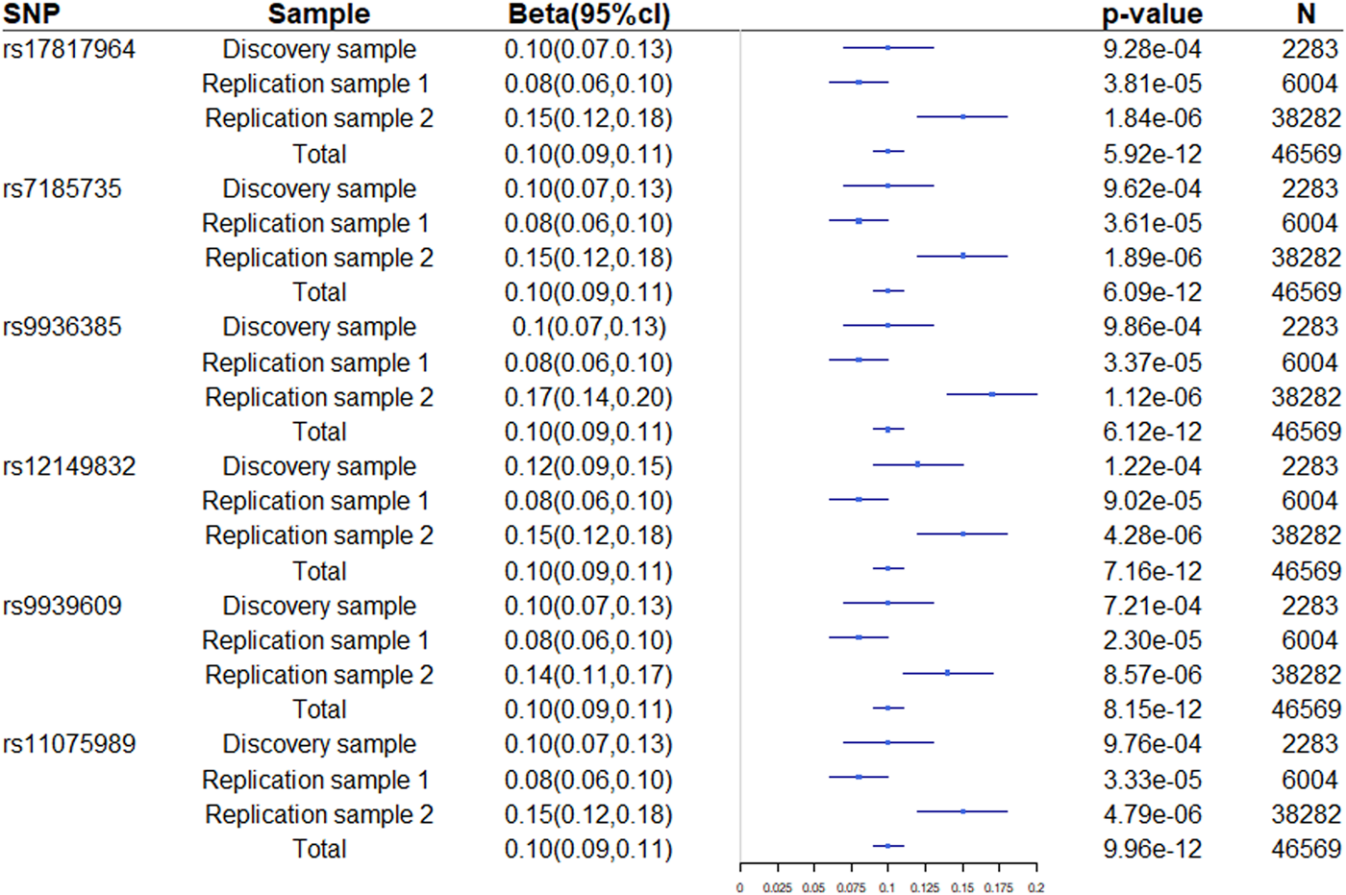
Forest plot of SNPs with combined p-value less than 1 × 10^−11^. Regression coefficient (beta) and its 95% confidence interval (CI) are presented in untransformed estimates from individual studies. “Total” refers to the combined meta-analysis.

**Figure 5:**
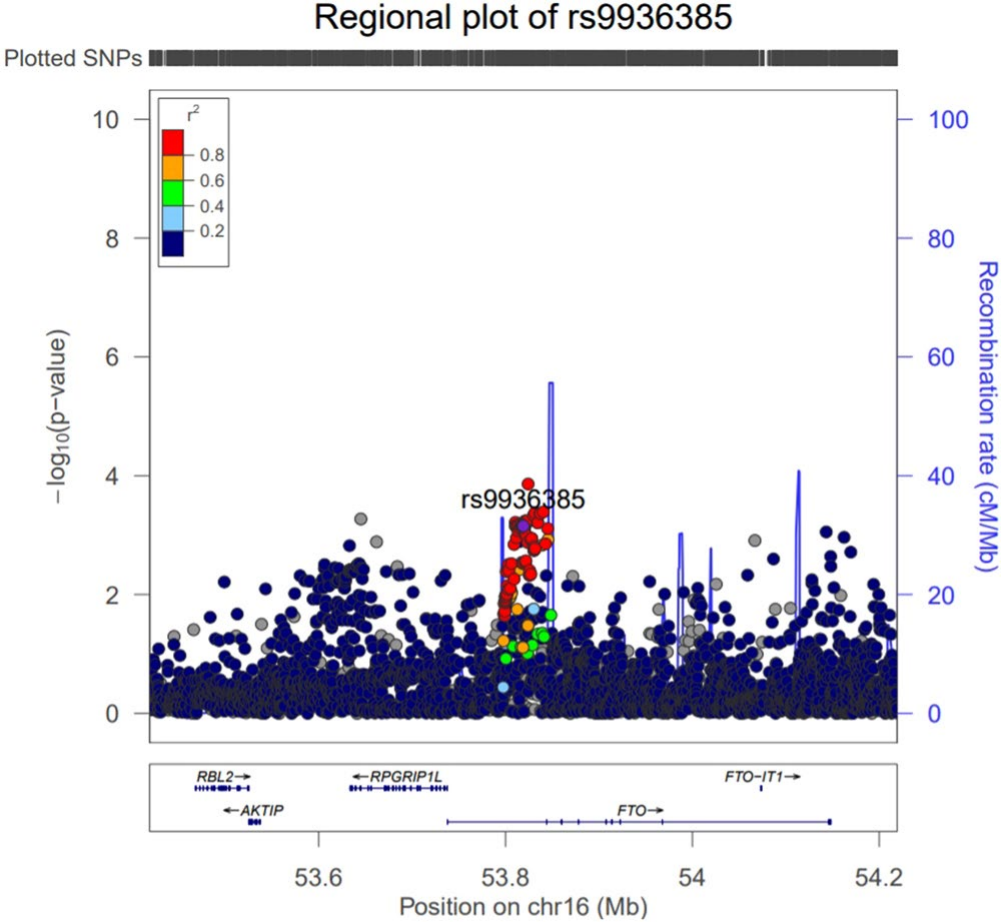
Regional plot of FTO generated using Locus Zoom.

We identified 29 SNPs located in the FTO gene demonstrated associations with LMI in the discovery sample (*p* < 10^−2^). LD analysis showed that these 29 SNPs were in LD (r^2^ ≥ 0.91) and were located within two LD blocks (Figure 3). These SNPs were replicated in independent Caucasian replication samples (Table 2). Meta-analysis *p*-values ranging from 5.92 × 10^−12^ to 1.69 × 10^−9^. SNP rs17817964 is the most significant SNP with combined *p* = 5.92 × 10^−12^ in discovery sample and two replication samples of Caucasian. There are 6 SNPs with *p* value less than 1 × 10^−11^. Forest plot of SNPs with combined *p* < 1 × 10^−11^ was drawn in Figure 4. Regional plot of the gene FTO was drawn by LocusZoom in Figure 5.

**Table 2:** Significant association results for SNPs.

SNP	position	region	Allele^a^	Discovery sample (LMI)				Replication sample 1 (LBM)				Replication sample 2 (LBM)				Combined *p*
				MAF	Beta	*p*	N	MAF	Beta	*p*	N	MAF	Beta	*p*	N	
rs17817964	53794154	Intron	C/T	0.60	−0.10	9.28 × 10^−4^	2,207	0.60	−0.08	3.81 × 10^−5^	6,004	0.60	−0.15	1.84 × 10^−6^	38,282	5.92 × 10^−12^
rs7185735	53788739	Intron	A/G	0.60	−0.10	9.62 × 10^−4^	2,207	0.59	−0.08	3.61 × 10^−5^	6,004	0.60	−0.15	1.89 × 10^−6^	38,285	6.09 × 10^−12^
rs9936385	53785257	Intron	C/T	0.40	0.10	9.86 × 10^−4^	2,207	0.41	0.08	3.37 × 10^−6^	6,004	0.39	0.17	1.12 × 10^−6^	36,349	6.12 × 10^−12^
rs12149832	53808996	Intron	A/G	0.41	0.12	1.22 × 10^−4^	2,207	0.41	0.08	9.02 × 10^−5^	6,004	0.42	0.15	4.28 × 10^−6^	38,171	7.16 × 10^−12^
rs9939609	53786615	Intron	A/T	0.40	0.10	7.21 × 10^−4^	2,207	0.41	0.08	2.30 × 10^−5^	6,004	0.40	0.14	8.57 × 10^−6^	38,286	8.15 × 10^−12^
rs11075989	53785965	Intron	C/T	0.60	−0.10	9.76 × 10^−4^	2,207	0.59	−0.08	3.33 × 10^−5^	6,004	0.60	−0.15	4.79 × 10^−6^	38,337	9.96 × 10^−12^
rs11075990	53785981	Intron	A/G	0.60	−0.10	9.76 × 10^−4^	2,207	0.59	−0.08	3.33 × 10^−5^	6,004	0.60	−0.15	4.61 × 10^−6^	38,337	1.02 × 10^−11^
rs3751812	53784548	Intron	G/T	0.60	−0.10	1.06 × 10^−3^	2,207	0.59	−0.08	3.34 × 10^−5^	6,004	0.60	−0.15	4.81 × 10^−6^	38,325	1.10 × 10^−11^
rs8050136	53782363	Intron	A/C	0.40	0.10	1.10 × 10^−3^	2,207	0.42	0.08	2.89 × 10^−5^	6,004	0.40	0.14	6.64 × 10^−6^	38,237	1.17 × 10^−11^
rs9935401	53782926	Intron	A/G	0.40	0.10	1.01 × 10^−3^	2,207	0.41	0.08	3.53 × 10^−5^	6,004	0.40	0.14	5.26 × 10^−6^	38,338	1.28 × 10^−11^
rs8051591	53782840	Intron	A/G	0.60	−0.10	1.01 × 10^−3^	2,207	0.59	−0.08	3.54 × 10^−5^	6,004	0.60	−0.14	5.62 × 10^−6^	38,338	1.35 × 10^−11^
rs17817449	53779455	Intron	G/T	0.40	0.10	1.01 × 10^−3^	2,207	0.41	0.08	3.62 × 10^−5^	6,004	0.40	0.14	5.79 × 10^−6^	38,338	1.44 × 10^−11^
rs8043757	53779538	Intron	A/T	0.60	−0.10	1.01 × 10^−3^	2,207	0.59	−0.08	3.61 × 10^−5^	6,004	0.60	−0.14	5.86 × 10^−6^	38,338	1.45 × 10^−11^
rs9923233	53785286	Intron	C/G	0.40	0.10	9.86 × 10^−4^	2,207	0.41	0.08	3.39 × 10^−5^	6,004	0.41	0.14	9.59 × 10^−6^	38,242	1.71 × 10^−11^
rs17817288	53773852	Intron	A/G	0.51	−0.09	2.44 × 10^−3^	2,207	0.50	−0.08	3.24 × 10^−5^	6,004	0.51	−0.14	8.61 × 10^−6^	38,016	5.44 × 10^−11^
rs1558902	53769662	Intron	A/T	0.41	0.09	2.40 × 10^−3^	2,207	0.42	0.08	4.75 × 10^−5^	6,004	0.41	0.14	7.52 × 10^−6^	38,261	5.54 × 10^−11^
rs7202116	53787703	Intron	A/G	0.60	−0.10	9.63 × 10^−4^	2,207	0.59	−0.08	3.68 × 10^−5^	6,004	0.60	−0.16	1.87 × 10^−5^	28,232	5.69 × 10^−11^
rs1421085	53764042	Intron	C/T	0.41	0.09	2.30 × 10^−3^	2,207	0.42	0.08	4.87 × 10^−5^	6,004	0.41	0.14	7.72 × 10^−6^	38,254	5.73 × 10^−11^
rs9930506	53796553	Intron	A/G	0.56	−0.10	7.99 × 10^−4^	2,207	0.57	−0.07	3 × 10^−4^	6,004	0.56	−0.13	4.66 × 10^−5^	37,911	4.96 × 10^−10^
rs9922619	53797859	Intron	G/T	0.56	−0.10	8.76 × 10^−4^	2,207	0.57	−0.07	3 × 10^−4^	6,004	0.56	−0.13	5.48 × 10^−5^	38,038	7.52 × 10^−10^
rs9922708	53797234	Intron	C/T	0.56	−0.10	8.90 × 10^−4^	2,207	0.57	−0.07	3 × 10^−4^	6,004	0.56	−0.13	5.70 × 10^−5^	38,038	7.69 × 10^−10^
rs9932754	53796579	Intron	C/T	0.44	0.10	1.04 × 10^−3^	2,207	0.43	0.07	3 × 10^−4^	6,004	0.44	0.13	5.98 × 10^−5^	38,038	9.12 × 10^−10^
rs9930501	53796540	Intron	A/G	0.56	−0.10	1.04 × 10^−3^	2,207	0.57	−0.07	3 × 10^−4^	6,004	0.56	−0.13	6.31 × 10^−5^	38,037	9.62 × 10^−10^
rs9931494	53793267	Intron	C/G	0.58	−0.09	3.38 × 10^−3^	2,207	0.58	−0.07	2 × 10^−4^	6,004	0.58	−0.13	2.79 × 10^−5^	38,118	1.08 × 10^−9^
rs7201850	53787950	Intron	C/T	0.58	−0.09	3.37 × 10^−3^	2,207	0.58	−0.07	2 × 10^−4^	6,004	0.58	−0.13	2.89 × 10^−5^	38,118	1.12 × 10^−9^
rs9941349	53791576	Intron	C/T	0.58	−0.09	3.41 × 10^−3^	2,207	0.58	−0.07	2 × 10^−4^	6,004	0.58	−0.13	2.71 × 10^−5^	38,106	1.15 × 10^−9^
rs8044769	53805223	Intron	C/T	0.52	0.10	5.37×10^−4^	2,207	0.52	0.07	5 × 10^−4^	6,004	0.52	0.13	5.79 × 10^−5^	38,303	1.20 × 10^−9^
rs9922047	53772368	Intron	C/G	0.49	−0.08	5.85 × 10^−3^	2,207	0.48	−0.08	9.21 × 10^−5^	6,004	0.48	−0.13	6.34 × 10^−5^	38,164	1.37 × 10^−9^
rs11075987	53781249	Intron	G/T	0.51	0.09	3.09 × 10^−3^	2,207	0.51	0.07	2 × 10^−4^	6,004	0.51	0.13	3.97 × 10^−5^	38,185	1.69 × 10^−9^

^a^The first allele represents the minor allele of each marker.

**Figure 6:**
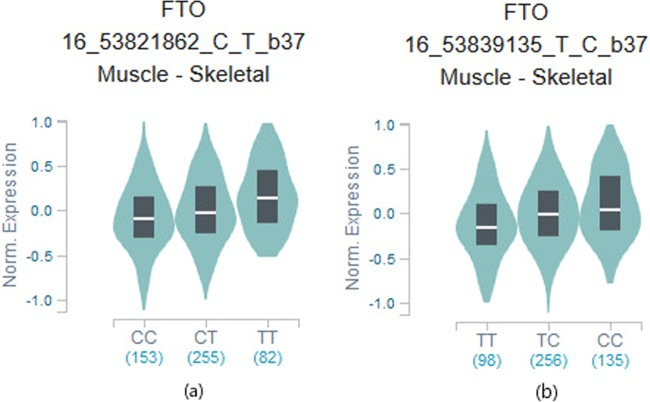
(**a**) Box plot of eQTL rs7201850. (**b**) Box plot of eQTL rs8044769 Box plot of eQTL variant results (p = 1 × 10^−5^): rs7201850-muscle skeletal, rs8044769-muscle skeletal. These variants showed significant eQTL in their minor allele.

**Figure 7:**
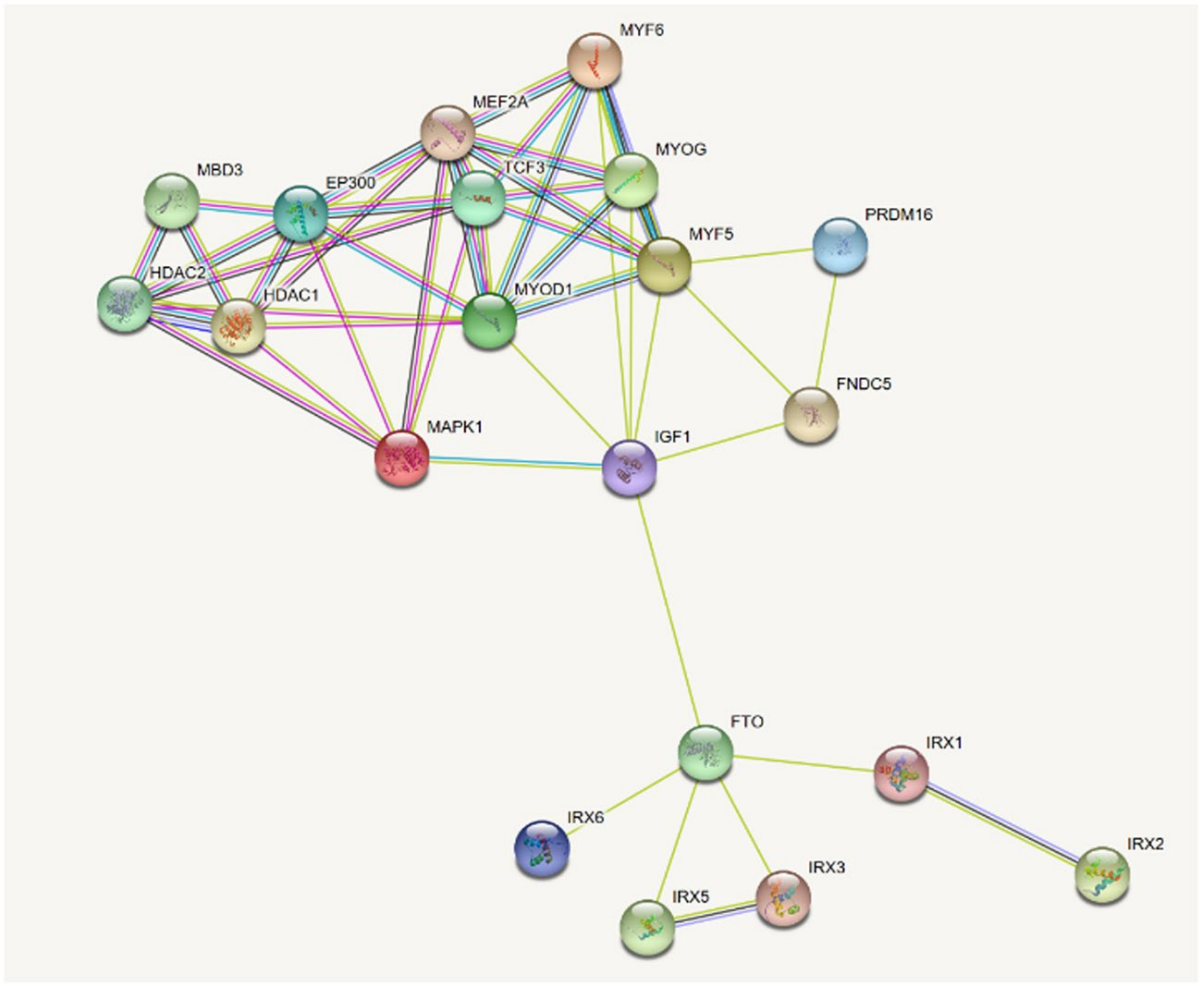
Interaction network for FTO. Proteins in the interaction network were represented with nodes, while the interaction between any two proteins therein was represented with an edge. Line color indicates the type of interaction evidence including known interactions, predicted interactions and other. These interactions contain direct (physical) and indirect (functional) interactions, derived from numerous sources such as experimental repositories, computational prediction methods.

The results of biological functional annotation using HaploReg v4.1, Regulome DB and GTEx are performed in Table 3. 25 SNPs may locate in a strong enhancer region marked by peaks of several active histone methylation modifications (H3K27ac, H3K9ac, H3K4me1 and H3K4me3). SNP rs17817288 (discovery *p* = 2.44 × 10^−3^, combined *p* = 5.44 × 10^−11^) occupies promoter histone marks in muscle satellite cultured cells. It was predicted to have enhancer activity by chromatin states, H3K4me1 and H3K27ac marks in skeletal muscle myoblasts cells and H3k4me1 marks in muscle satellite cultured cells. Besides it has promoter activity, implied by H3K4me3 and H3K9ac in muscle satellite cultured cells and H3K9ac in HSMM skeletal muscle myoblasts cells. Among the 29 SNPs evaluated with Regulome DB, 7 had no data. Of the 22 SNPs for which Regulome DB provided a score, 2 had a score of <3 (likely to affect the binding) including rs17817964 and rs7202116 with Regulome DB score = 2b respectively. Analyses using GTEx data reveal 11 SNPs of our GWAS results have strong signals of cis-eQTL for FTO gene in skeletal muscle tissue (*p* < 1 × 10^−4^). SNPs rs7201850 and rs8044769 were deposited in the GTEx eQTL database as a cis-eQTL for FTO in skeletal muscle with the same direction of effect (*p* = 1 × 10^−5^, Figure 6). Gene-gene interaction networks shows there are some connections between FTO and IGF-1, myogenic regulatory factors (MRFs: MYF5, MYOD1, MYOG, and MYF6) and IRX3, implying that FTO may play an important role in muscle development (Figure 7).

**Table 3:** Biological function annotation.

Variant	Promoter	Enhancer	DNAse	Proteins	Motifs	GENCODE	dbSNP	Regulome	eQTL
	histone marks	histone marks		Bound	Changed	Genes	func annot	DB score^a^	*p* –value^b^
rs17817964		5 tissues		GATA3	5 altered motifs	FTO	intronic	2b	—
rs7185735		6 tissues			Gcm1,Mef2	FTO	intronic	—	—
rs9936385		8 tissues	17 tissues		HDAC2,Pax-5	FTO	intronic	5	—
rs12149832	BRN	11 tissues	BRST		XBP-1	FTO	intronic	6	4 × 10^−5^
rs9939609		BRST			Nanog,Pou5f1	FTO	intronic	—	—
rs11075989		BRST, FAT, LNG			6 altered motifs	FTO	intronic	6	—
rs11075990		BRST, FAT, LNG			Nkx6-1,Pou4f3,Pou6f1	FTO	intronic	6	—
rs3751812		12 tissues	7 tissues		Mrg,TBX5,Tgif1	FTO	intronic	3a	—
rs8050136		8 tissues	BRST,CRVX,BRST	P300	6 altered motifs	FTO	intronic	4	—
rs9935401		BRST, SKIN			Cdx,HES1	FTO	intronic	—	—
rs8051591		BRST, CRVX, SKIN	BRST		6 altered motifs	FTO	intronic	6	—
rs17817449		11 tissues	5 tissues		4 altered motifs	FTO	intronic	5	4 × 10^−5^
rs8043757		11 tissues	BRST,SKIN		Evi-1	FTO	intronic	5	—
rs9923233		8 tissues	14 tissues		8 altered motifs	FTO	intronic	5	—
rs17817288	MUS, LIV	17 tissues	6 tissues	FOXA1,FOXA2,TCF4	8 altered motifs	FTO	intronic	5	—
rs1558902	LNG	16 tissues	GI		GATA	FTO	intronic	—	5 × 10^−5^
rs7202116		6 tissues	BLD	MAFF,MAFK	7 altered motifs	FTO	intronic	2b	—
rs1421085	LIV	14 tissues	LIV,VAS		Arid3a,HNF6	FTO	intronic	5	3 × 10^−5^
rs9930506					Irx	FTO	intronic	6	5 × 10^−5^
rs9922619					6 altered motifs	FTO	intronic	6	—
rs9922708		HRT	HRT		HEN1,Pbx-1,TAL1	FTO	intronic	6	4 × 10^−5^
rs9932754					6 altered motifs	FTO	intronic	6	5 × 10^−5^
rs9930501					Nanog,SRF	FTO	intronic	—	4 × 10^−5^
rs9931494		FAT			11 altered motifs	FTO	intronic	6	—
rs7201850		7 tissues			Foxo,RORalpha1	FTO	intronic	—	1 × 10^−5^
rs9941349		BRN				FTO	intronic	—	—
rs8044769		8 tissues	6 tissues	JUND,CJUN	4 altered motifs	FTO	intronic	4	1 × 10^−5^
rs9922047	FAT	13 tissues				FTO	intronic	5	—
rs11075987	LNG	14 tissues	8 tissues	STAT3			intronic	4	5 × 10^−5^

^a^Prediction for SNP from Regulome DB with score=2b: TF binding + any motif + DNAse Footprint + DNase peak; score = 3a: TF binding + any motif + DNase peak; score = 4: TF binding + DNase peak; score = 5: TF binding or DNase peak; score = 6: other.

^b^The GWAS SNPs are the significant eQTLs for FTO in skeletal muscle from GTEx.

## Discussion

In this study, we have performed a GWAS in 2,207 Caucasian subjects and replicated this result in three replication samples including 6,004 unrelated Caucasian from FHS and 38,292 unrelated Caucasian^30^. We identified 29 SNPs in FTO gene associated with LMI then we performed the potential biological function annotation of SNPs. In this study, FTO is suggested to be associated with lean mass.

FTO gene encodes a 2-oxoglutarate (2-OG) Fe(II) dependent nucleic acid demethylase belonging to the AlkB-related non-heme dioxygenase (Fe(II-)- and 2-oxoglutarate-dependent dioxygenases) superfamily of proteins. In the previous studies, FTO was identified to be related to increased risk of obesity and a T2D incurrence^17,40^. Studies have shown that the expression of FTO protein in lean mass and adipose tissue is related to the oxidation rate of whole body substrate. With the increase of age, the body’s carbohydrate oxidation rate decreases, the fat oxidation rate increases, and at the meanwhile FTO protein expression increases in adipose but that decreases in skeletal muscle mass^41^. Loos *et al*. have shown that homozygous Fto −/− mice have postnatal growth retardation, obviously decreasing in adipose tissue, and LBM^40^. According to the studies of athletes the T-allele of FTO gene rs9939609 is associated with increased lean mass for the elite rugby athletes, and for combat sports athletes the A-allele is related with decreased slow-twitch muscle fibers^29,42^. AMPK (AMP-activated protein kinase) is an essential part of skeletal muscle lipid metabolism and is the major cellular energy sensor. In skeletal muscle cells AMPK reduces mRNA m6A methylation and lipid accumulation by FTO-dependent demethylation at the molecular level^43^.

We found there are some connections between FTO and IRX3 in the gene-gene interaction networks. To evaluate phenotypic consequence associated with muscle of the FTO and IRX3 genes, we surveyed mouse knockout models. We searched the international mouse phenotyping consortium (IMPC) database (http://www.mousephenotype.org/) as well as the literature about knockout models related to muscle phenotypes. In IMPC database, of two genes that have results of DXA scan, FTO has abnormal body weight and compared to normal controls IRX3 has abnormal lean body mass in knockout mice (*p* < 0.05). According to the studies of FTO knockout mice, mouse have reduced fat mass as well as lean mass which is independent of its effect on food intake^22,23^. Besides, FTO-deficient mice showed skeletal muscle development was damaged^28^. Some vitro and vivo experiments have shown during myoblasts differentiation FTO expression increased and FTO silencing inhibited myoblasts differentiation^28^. Homozygote FTO deficiency mice have decreased body weight including decreased body size, abnormal body weight and decreased total tissue weight in the IMPC database. Because there is a greater browning of white adipose tissues, IRX3 knockout mice need more energy to expend, particularly at night. Recent findings show brown fat is associated with muscle developmental precursor Myf5^44,45^. Homozygote IRX3 deficiency mice have decreased LBM and increased total body fat mass in the IMPC database.

## Conclusion

In summary, we identified the FTO gene were significantly association with lean mass in the Caucasian subjects. However, the clear function between FTO gene and lean mass is still unknown that needs more researches to reveal.

## Data Availability

The datasets used and/or analyzed during the current study are available from the corresponding author on a reasonable request.
